# Red cell distribution width as a novel marker for predicting high-risk from upper gastro-intestinal bleeding patients

**DOI:** 10.1371/journal.pone.0187158

**Published:** 2017-11-02

**Authors:** Kyeong Ryong Lee, Sang O. Park, Sin Young Kim, Dae Young Hong, Jong Won Kim, Kwang Je Baek, Dong Hyuk Shin, Young Hwan Lee

**Affiliations:** 1 Department of Emergency Medicine, School of Medicine, Konkuk University, Konkuk University Medical Center, Seoul, Republic of Korea; 2 Department of Emergency Medicine, Kangbuk Samsung Hospital, Sungkyunkwan University School of Medicine, Seoul, Republic of Korea; 3 Department of Emergency Medicine, Soonchunhyang University Bucheon Hospital, Bucheon, Korea; University Hospital Llandough, UNITED KINGDOM

## Abstract

**Background:**

In upper gastrointestinal bleeding (UGIB) patients, early risk stratification allows appropriate therapy that may be helpful for reducing morbidity and mortality.

**Objectives:**

to evaluate the efficacy of red-cell distribution width (RDW) for prediction of high-risk in UGIB patients.

**Methods:**

We conducted a clinical retrospective observational study based on data for UGIB patients from 2012 to 2013. The primary outcome was the high-risk UGIB, defined as those who required urgent intervention and/or 30-days mortality. RDW was categorized into four quartiles: Q1 (≤12.8%), Q2 (12.9–14.4%), Q3 (14.5–16.5%), and Q4 (≥16.6%), and multivariable analysis was performed after adjustment of multiple other risk factor. We also evaluated the efficacy of addition of RDW scores to the Pre-endoscopic Rockall Score (PRS) and the Glasgow Blatchford Score (GBS) scoring system.

**Results:**

Of 360 UGIB patients, 229 (63.6%) were high risk. In multivariable analysis, Q3 and Q4 were strongly associated with high risk; odds ratio (95% Confidence Interval) was 3.144 (1.250–7.905) and 4.182 (1.483–11.790) respectively (all *p* < 0.05). For lower GBS score group (≤ 6), the incidence of high risk was higher in Q4 (30%) and Q3 (20%) than in Q2 (12.5%) and Q1 (11.4%). For lower PRS group (≤ 2), the incidence of high-risk was higher in Q4 (73.7%) and Q3 (57.1%) than in Q1 (35.4%). Receiver operating characteristic analysis showed higher discrimination power in PRS + RDW (Area Under Curve [AUC] = 0.749) than PRS (AUC = 0.715) alone (*p =* 0.036). Otherwise GBS + RDW (AUC = 0.873) did not show a significant higher discrimination power than the GBS (AUC = 0.864) alone (p = 0.098).

**Conclusions:**

For UGIB patients, a high RDW (≥ 14.5%) was strongly associated with high risk UGIB. In practice, the combination of RDW with the PRS scoring indexes may increase the accuracy of risk stratification.

## Introduction

Acute upper gastrointestinal bleeding (UGIB) is a commonly encountered cause of admission to hospital. Patients presenting with acute UGIB have a wide range of clinical courses. Some cases of UGIB are self-limiting and do not require any significant treatment; however, urgent treatment, such as blood transfusion, endoscopic therapy and/or surgical therapy, is occasionally required in patients with severe UGIB. Despite 70–80% of UGIB cases being self-limiting, its mortality has been reported to be 6–13% [1–3].

In UGIB patients, early risk stratification allows appropriate therapy that may be helpful for reducing morbidity and mortality. Multiple clinical factors, including old age, unstable vital signs, melena, and various comorbid illnesses, together with laboratory markers such as low hemoglobin (Hg) and elevated blood urea nitrogen (BUN) levels, are known to be associated with a high risk of morbidity and mortality [4]. Risk stratification principally depends on clinical decision, and universal risk stratification method was not established because there were multifactorial associations with the modality and mortality in UGIB patients.

Red-cell distribution width (RDW), which is a routine component of complete blood counts (CBC), represents the variability in size of circulating erythrocytes. Traditionally, this measure has been used to differentiate the etiology of anemia [5]. However, there is a widespread feeling that RDW may be a useful marker for predicting morbidity and mortality in various critical diseases [6–10]. The details of the relationship between an elevated RDW and poor outcomes have not been identified, although recent reports have revealed that RDW has an independent, linear relationship with recurrent or massive bleeding in critical conditions, including post-percutaneous coronary intervention, intracranial hematoma and multiple trauma patients [11–13].

Considering the fact that some UGIB patients who suffer ongoing bleeding have a high risk of mortality, the possibility of using early RDW levels to allow risk stratification of patients with acute UGIB can be confidently hypothesized. The aim of this study was to evaluate the efficacy of early RDW to predict patients with high-risk UGIB, including those who require urgent therapy and those with a higher risk of morbidity and mortality.

## Materials and methods

This retrospective, single-center observational study was conducted at Konkuk University Medical Centre, Seoul, South Korea. The Konkuk University Hospital Institutional Review Board approved the study protocol (KUH-1260014), and all data were collected from electronic medical records. All patients' data were fully anonymized before we accessed them. Our Institutional Review Board reviewed this and confirmed it.

During the 2-year study period (Jan 2012 to Dec 2013), we enrolled all patients over 18 years of age who were diagnosed in the emergent department with UGIB, which was defined as the presence of hematemesis/coffee-ground vomitus or melena. Exclusion criteria were a known hematologic disease, refusal of the laboratory study, further treatment or requested transport to another treatment facility and pregnant woman. Routine whole blood tests, including CBC, electrolytes, BUN, creatinine, liver function tests and coagulation tests were performed for all UGIB patients. CBC included Hg, hematocrit, RDW and other markers. All markers were measured in all UGIB patients within 1 h of arrival at the emergency department.

We obtained detailed clinical data including the patients’ age, sex, active medication (current medication history of NSAIDS, Aspirin or Warfarin),comorbidities such as congestive heart failure, coronary artery disease, renal failure (patients diagnosed with acute kidney injury or chronic kidney disease), liver failure (patients diagnosed with acute liver failure or chronic liver cirrhotic patients except for a case where liver function was compensated) and metastatic cancer; their initial vital signs, including systolic blood pressure (SBP) and pulse rate; their history of syncope; and the presence of melena or hematemesis. Data about the results of treatment that were obtained included transfusions, re-bleeding, endoscopic findings, hemostatic therapy including the endoscopic adrenergic injection, endoscopic clipping, endoscopic thermo-coagulation, band ligation, trans-arterial embolization and surgery, and mortality. In addition, we used two clinical decision-making algorithms popular in the emergency department: the Pre-endoscopic Rockall Score (PRS) and the Glasgow Blatchford Score (GBS). The PRS is calculated using age, pulse rate, SBP and comorbidity [14] the GBS is calculated using BUN, Hg, SBP, pulse rate, melena, syncope, hepatic disease and cardiac failure [15]. Patients were categorized into the following four groups by RDW quartile: 1) Q1 (< 12.8%), 2) Q2 (12.9–14.4%), 3) Q3 (14.5–16.5%), and 4) Q4 (> 16.6%).

The high-risk group was defined as those who were treated by urgent blood transfusion, endoscopic therapy or surgery because of continued bleeding, as well as those who developed serious in-hospital complications including shock, re-bleeding or death. All patients who were not treated with specific interventions were classified as low risk. For determination of the variables associated with high-risk patients, multivariate logistic regression analysis was performed. In addition, we constructed receiver operating characteristic (ROC) curves, and the areas under the curves (AUC) and 95% confidence intervals (CI) was calculated to compare the discriminatory power of PRS, GBS and RDW level for predicting high-risk patients. All data were processed and all statistical analyses were performed using SPSS Statistics 17.0 (SPSS Inc., Chicago, IL) and web-based free-ware R 3.0 statistics program. A two-sided *p-*value < 0.05 was considered significant.

## Results

### Baseline clinical data

During the study period, 394 patients were diagnosed with UGIB, among which, 34 were excluded because they refused treatment and/or laboratory evaluation (No = 6) or requested transportation to another facility (No = 16). Twelve patients with hematologic disease were excluded. Lastly, 360 patients met our study criteria. Table 1 shows the basic characteristics and outcomes of the study population.

**Table 1:** Data of demographic and outcomes in the upper gastro-intestinal bleeding patients.

Characteristics	Value
Age, mean (SD)	58.6 ± 17.1
Age ≥ 60 years, No. (%)	160 (44.4)
Male, No. (%)	254 (70.6)
Active medication, No (%)^a^	51 (14.2)
NSAIDS	3 (0.8)
Antiplatelet	38 (10.6)
Anticoagulant	13 (3.6)
Comorbidities, No. (%) ^a^	201 (55.8)
Congestive heart failure, No. (%)	16 (4.4)
Coronary artery disease, No. (%)	24 (6.7)
Renal failure, No. (%)	54 (15.0)
Liver failure, No. (%)	132 (36.7)
Metastatic cancer, No. (%)	32 (8.9)
Initial findings	
SBP < 100 mmHg, No. (%)	78 (21.7)
Pulse rate > 100/min, No. (%)	147 (40.8)
Melena, No. (%)	246 (68.3)
Syncope, No. (%)	31 (8.6)
Cause of UGIB ^a^	
Ulcer, No. (%)	140 (38.9)
Variceal, No. (%)	93 (25.8)
Mallory-Weiss, No. (%)	46 (12.8)
Gastroduodenal ^b^, No. (%)	43 (11.9)
Neoplastic, No. (%)	27 (7.5)
Unknown, No. (%)	11 (3.1)
Treatment^a^	
Non-intervention, No. (%)	131 (36.4)
Blood transfusion, No. (%)	223 (61.9)
Hemostatic therapy ^c^, No. (%)	83 (23.1)
In-hospital mortality, No. (%)	21 (5.8)

SD: standard deviation; SBP: systolic blood pressure

^a^ duplicated

^b^ This includes esophageal ulcer, esophagitis, gastritis,bulbitis and erosions

^c^ This includes endoscopic adrenergic injection, endoscopic clip, endoscopic thermo-coagulation, band ligation, trans-arterial embolization and surgery

Of the 360 patients who were enrolled in the study, endoscopy was performed in 352 except for two patients who died in the ED before the endoscopy and six who were denied an endoscopy. Among patients who underwent an endoscopy, a total of 18 patients (5.0%) were treated using adrenergic injection only and five patients (1.4%) were treated using endoscopic clips only. Twenty-one patients (5.8%) were treated using multi endoscopic therapies (combination of adrenergic injection, endoscopic clipping, and/or thermo-coagulation). For variceal bleeding controls, 37 band ligations (10.3%) were performed. For three patients who had refractory UGIB, trans-arterial embolization (No = 2) or emergency surgery (No = 1) were performed. Eighty-three patients were treated by hemostatic therapy with or without blood transfusion. In conclusion, a total of 229 patients (63.6%) was categorized as the high-risk group.

Compared with the low-risk patients, the high-risk patients were older and had a higher incidence of comorbidities and active medication. The initial findings indicated that a higher rate of low SBP (< 100 mmHg), high pulse rate (> 100/min), melena and syncope were observed in the high-risk UGIB group, and that they had higher BUN and RDW and lower Hg (Table 2). As for the cause of UGIB, variceal bleeding (71/93; 76.3%) showed a higher proportion of risk than non-variceal bleeding (158/267; 59.2%) (p = 0.004). Table 2 shows the distribution of all causes of UGIB between the low- and high-risk groups.

**Table 2:** Comparisons of baseline characteristics and clinical features between the high risk and low risk in the upper gastro-intestinal bleeding patients.

	Risk stratification		
	High risk (No = 229)	Low risk (No = 131)	p-value
Age, mean (SD)	61.8 15.1	52.9 ± 19.0	< 0.001
Male, No (%)	158 (69.0)	96 (73.3)	0.403
Comorbidities, No (%)	147 (73.1)	54 (26.9)	< 0.001
Active medication	41 (17.9)	10 (7.6)	0.007
Initial findings			
SBP < 100 mmHg, No (%)	69 (30.1)	9 (6.9)	< 0.001
Pulse rate > 100/min, No (%)	106 (46.3)	41 (31.3)	0.005
Melena, No (%)	141 (70.1)	60 (29.9)	< 0.001
Syncope, No (%)	25 (10.9)	6 (4.6)	0.050
Initial laboratory			
Hg, mean (SD)	8.1 ± 2.4	12.6 ± 2.5	< 0.001
BUN, median (IQR)	32.4 (22.5–44.6)	19.6 (13.9–35.7)	< 0.001
RDW, mean (SD)	15.7 ± 2.9	13.9 ± 2.1	< 0.001
Risk predicting score system			
GBS, median (IQR)	12.0 (10.0–13.0)	5.0 (1.0–9.0)	< 0.001
PRS, median (IQR)	3.0 (2.0–5.0)	1.0 (0.0–3.0)	< 0.001
Cause of UGIB ^a^			< 0.001
Ulcer, No. (%)	96 (41.9)	44 (33.6)	
Variceal, No. (%)	71 (31.0)	22 (16.8)	
Mallory-Weiss, No. (%)	10 (4.4)	36 (27.5)	
Gastroduodenal ^b^, No. (%)	20 (8.7)	23 (17.6)	
Neoplastic, No. (%)	25 (92.6)	2 (1.5)	
Unknown, No. (%)	7 (3.1)	4 (3.1)	

SD, standard deviation; SBP, systolic blood pressure; Hg, hemoglobin; BUN, blood urea nitrogen; RDW, Red cell distribution width; IQR, Interquartile ranges 25% -75%; GBS, Glasgow Blatchford Score; PRS, Pre-endoscopic Rockall Score

### The relationship between the RDW values and high risk UGIB

Table 3 outlines the patients’ characteristics and clinical data by RDW quartile. Higher RDW quartiles were significantly associated with increased age, a higher incidence of comorbidities, higher incidence of active medication and a decrease in Hg. Higher RDW quartiles (especially Q3 and Q4) also included a higher frequency of high-risk UGIB and increased GBS and PRS scores compared with the lower quartile RDW groups.

**Table 3:** Comparisons of baseline characteristics and clinical features among the four quarter red-cell distribution width (RDW) groups.

	Q1 (No = 94)	Q2 (No = 89)	Q 3 (No = 89)	Q4 (No = 88)	*P*-value
Age, mean (SD)	45.1 ±	62.1 ± 15.7	65.7 ± 15.6	58.6 ± 11.4	< 0.001
Male, No (%)	70 (74.5)	66 (74.2)	58 (65.2)	60 (68.2)	0.435
Comorbidities, No (%)	20 (21.3)	46 (51.7)	64 (71.9)	71 (80.7)	< 0.001
Congestive heart failure, No. (%)	1 (1.1)	4 (4.5)	5 (5.6)	6 (6.8)	0.263
Coronary artery disease, No. (%)	1 (1.1)	8 (9.0)	8 (9.0)	7 (8.0)	0.089
Renal failure, No. (%)	6 (6.4)	19 (21.3)	19 (21.3)	10 (11.4)	0.007
Liver failure, No. (%)	13 (13.8)	24 (27.0)	40 (44.9)	55 (62.5)	< 0.001
Metastatic cancer, No. (%)	2 (2.1)	3 (3.4)	5 (5.6)	7 (8.0)	0.265
Active medication	5 (5.3)	14 (15.7)	18 (20.2)	14 (15.9)	0.028
Initial findings					
SBP < 100 mmHg, No (%)	15 (16.0)	15 (16.9)	25 (28.1)	23 (26.1)	0.101
Pulse rate > 100/min, No (%)	37 (39.4)	34 (38.2)	36 (40.4)	40 (45.5)	0.772
Melena, No (%)	56 (59.6)	64 (71.9)	64 (71.9)	62 (70.5)	0.206
Syncope, No (%)	9 (9.6)	8 (9.0)	7 (7.9)	7 (8.0)	0.971
Initial maboratory					
Hg, mean (SD)	12.4 ± 2.8	10.2 ± 3.0	8.7 ± 2.5	7.6 ± 2.5	< 0.001
BUN, median (IQR)	29.9 (15.9–37.7)	30.7 (20.7–44.5)	21.8 (21.8–44.3)	25.3 (14.9–39.6)	0.007
GBS, median (IQR)	7.0 (1.0–11.0)	9.5 (6.0–12.0)	11.0 (9.0–13.0)	11.0 (9.3–14.0)	< 0.001
PRS, median (IQR)	1.0 (0.0–2.0)	2.0 (1.0–4.0)	4.0 (3.0–5.0)	4.0 (3.0–4.0)	< 0.001
High risk, No (%)	37 (39.4)	51 (57.3)	69 (77.5)	72 (81.8)	< 0.001
30-days mortality, No (%)	0 (0)	3 (3.4)	7 (7.9)	11 (12.5)	0.002

Q1, first quarter group (RDW ≤ 12.8%); Q2: second quarter group (RDW 12.9–14.4%); Q3: third quarter group (RDW 14.5–16.5%); Q4: fourth quarter group (RDW ≥16.6%); SD, standard deviation; SBP, systolic blood pressure; Hg, hemoglobin; BUN, blood urea nitrogen; IQR, Interquartile ranges 25% -75%; GBS, Glasgow Blatchford Score; PRS, Pre-endoscopic Rockall Score

In multivariate logistic regression analysis, a significant increase in the adjusted odd ratio (OR) of high-risk UGIB was found in RDW Q3 (OR: 3.144; 95% CI: 1.250–7.905) and Q4 (OR: 4.182; 95% CI: 1.483–11.790) compared with Q1. For other biomarkers, lower Hg (< 10.0 ng/ml) and higher BUN (> 25) were significantly associated with high-risk UGIB (OR: 10.805 and 12.751, respectively) (Table 4).

**Table 4:** Univariable and multivariable logical regression analysis of factors for high risk upper-gastrointestinal bleeding patients.

	Univariate			Multivariate		
	Odd ratio	Confidence Interval	p—value	Odd ratio	Confidence Interval	p–value
Old age (>60)	1.832	1.177–2.851	0.007	1.995	1.000–3.982	0.050
Male	1.233	0.764–1.987	0.391			
Comorbidity	2.556	1.646–3.971	< 0.001	0.978	0.502–1.905	0.948
Active medication	2.639	1.274–5.465	0.009	1.368	0.531–3.524	0.517
Clinical feature						
SBP< 100 mmHg	5.846	2.807–12.173	< 0.001	6.019	2.471–14.661	< 0.001
pulse rate > 100	1.892	1.204–2.971	0.006	2.744	1.460–5.157	0.002
Melena	3.059	1.927–4.853	< 0.001	2.259	1.185–4.307	0.013
Syncope	2.553	1.019–6.396	0.045	2.472	0.771–7.990	0.128
Initial laboratory						
Hg < 10	14.080	8.262–23.995	< 0.001	10.805	5.436–21.477	< 0.001
RDW Q1 (< 12.8%)	Baseline					
Q2 (12.9–14.4%)	2.068	1.147–3.728	0.016	1.628	0.726–3.653	0.237
Q3 (14.5–16.5%)	5.315	2.782–10.153	< 0.001	3.144	1.250–7.905	0.015
Q4 (> 16.6%)	6.932	3.506–13.707	< 0.001	4.182	1.483–11.790	0.007
BUN < 10	Baseline					
10–25	2.484	0.838–7.363	0.101	6.405	1.285–31.929	0.123
> 25	8.157	2.774–23.983	< 0.001	12.751	2.517–66.400	0.002

SBP: systolic blood pressure; Hg: hemoglobin; RDW: Red-cell distribution width; Q1, first quarter group (RDW ≤ 12.8%); Q2: second quarter group (RDW 12.9–14.4%); Q3: third quarter group (RDW 14.5–16.5%); Q4: fourth quarter group (RDW ≥ 16.6%);BUN: blood urea Nitrogen

We performed a subset analysis of the variceal bleedingand non-variceal bleeding patient groups. The mean RDW values in the high-risk group were higher than in the low-risk group in both variceal bleeding (17.0 ± 2.9 vs. 15.9 ± 1.9; p = 0.051) and non-variceal bleeding patients non-variceal bleeding patients (15.2 ± 2.7 vs. 13.5 ± 1.9; p < 0.001). In variceal bleeding patients (No = 93), RDW did not show a relationship with high-risk in a multivariate logistic regression analysis. In non-variceal bleeding patients (No = 267), a significant increase in the adjusted OR of high-risk UGIB was found in RDW Qnv4 (RDW ≥ 15.3%) (OR: 5.408; 95% CI: 1.624–18.004) compared with Qnv1 (≤ 12.6%) after adjusting for other factors in a multivariate logistic regression analysis (Table 5).

**Table 5:** Univariable and multivariable logical regression analysis of factors for high risk in non-variceal upper-gastrointestinal bleeding patients.

	Univariate			Multivariate		
	Odd ratio	Confidence Interval	p—value	Odd ratio	Confidence Interval	p–value
Old age (>60)	2.643	1.592–4.387	< 0.001	0.600	0.253–1.421	0.246
Male	0.924	0.543–1.575	0.773			
Comorbidity	2.097	1.257–3.497	0.005	0.615	0.279–1.355	0.615
Active medication	3.398	1.565–7.376	0.002	1.719	0.596–4.963	0.316
Clinical feature						
SBP< 100 mmHg	5.028	2.262–11.172	< 0.001	5.842	2.112–16.158	0.001
pulse rate > 100	1.772	1.054–2.977	0.031	2.815	1.317–6.018	0.008
Melena	4.038	2.337–6.978	< 0.001	2.421	1.115–5.256	0.025
Syncope	3.188	1.163–8.737	0.024	3.070	0.842–11.196	0.089
Initial laboratory						
Hg < 10	17.276	9.260–32.229	< 0.001	15.052	6.490–34.911	< 0.001
RDW Qnv1 (≤ 12.6%)	Baseline					
Qnv2 (12.7–13.6%)	2.090	1.040–4.201	0.038	1.571	0.611–4.037	0.348
Qnv3 (13.6–15.2%)	3.010	1.490–6.081	0.002	1.376	0.496–3.823	0.540
Qnv4 (≥ 15.3%)	9.284	4.105–20.997	< 0.001	5.408	1.624–18.004	0.006
BUN < 10	Baseline					
10–25	6.333	0.770–52.096	0.086			
> 25	23.283	2.869–188.958	0.003			

SBP: systolic blood pressure; Hg: hemoglobin; RDW: Red-cell distribution width; Qnv1, first quarter group (RDW ≤ 12.6%); Qnv2: second quarter group (RDW 12.7–13.6%); Qnv3: third quarter group (RDW 13.6–15.2%); Qnv4: fourth quarter group (RDW ≥ 15.3%); BUN: blood urea nitrogen

### Efficacy of RDW addition to the GBS and PRS systems

The process of categorizing GBS resulted in 88 patients (14.8%) being given a score of six or less; these patients were thought to be at lower risk of requiring interventions [4, 15]. In this group, the incidence of high-risk UGI was greater in Q3 and Q4 (20%; 2/10 and 30%; 3/10, respectively) than in Q1 and Q2 (11.4%; 5/44 and 12.5%; 3/24, respectively) (all *p* < 0.001). In the PRS scoring system, 164 patients (45.6%) had PRS scores of two or less; they were considered to have a good prognosis [4, 14]. The incidence of high-risk UGI was significantly greater in Q3 and Q4 (57.1%; 12/21 and 73.7%; 14/19, respectively) than in Q1 (35.4%; 28/79) (*p* = 0.017).

We used ROC curve analysis to compare the ability of GBS system, PRS system and the combination of GBS or PRS system plus the RDW score index (defined as zero for Q1 and Q2, 1 point for Q3 and 2 points for Q4 by reference to the data from the multivariable analysis) to discriminate high-risk UGIB. ROC curve analysis of GBS + RDW (AUC = 0.872) did not show a statistically significant increase in power of discriminating high-risk UGI patients than GBS alone (AUC = 0.864) (P = 0.098). Otherwise, PRS + RDW (AUC = 0.749) showed an increase in the power of discriminating high-risk UGI patients than PRS alone (AUC = 0.715) for discriminating high-risk UGI patients (p = 0.036) (Table 6).

**Table 6:** Test parameters of scoring systems and red blood cell distribution width (RDW) in prediction of high-risk upper gastrointestinal bleeding patients.

	AUC (95% C.I)	p-value*	Optimal value	Sensitivity (%)	Specificity (%)	PPV (%)	NPV (%)
RDW	0.714 (0.659–0.768)		14.5%	61.6	72.5	79.7	51.9
GBS	0.864 (0.823–0.905)	0.098	8	85.2	72.5	84.4	73.6
GBS + RDW	0.872 (0.833–0.912)		8	89.5	70.2	84.8	74.4
PRS	0.715 (0.662–0.769)	0.036	1	78.2	52.7	74.3	58.0
PRS + RDW	0.749 (0.698–0.800)		4	62.4	75.6	83.3	51.5

*DeLong’s test for two correlated ROC curves

AUC, Area Under Curve; PPV, Positive predictive value; NPV, Negative predictive value; GBS, Glasgow Blatchford Score; PRS, Pre-endoscopic Rockall Score

## Discussion

Early risk stratification of UGIB patients has been a challenging task in emergency department. RDW was easily checkable, cheap and fast achievable laboratory data. To the best of our knowledge, this is the first study to evaluate the role RDW as a predictor of high-risk in UGIB patients. This current study showed that higher RDW level (especially over 14.5%) is an independent predictor of high-risk UGIB after adjustment for various patient and clinical factors. RDW over 14.5% was associated with approximately over 3–4 fold higher incidence of high risk in UGIB in our adjusted models. In the PRS system, the combination of RDW with the PRS system can increase the accuracy of identification of those low-risk patients who do not need urgent therapy and improve the discriminatory power of risk stratification in UGIB patients.

RDW is a measurable parameter that represents the degree of heterogeneity of red blood cells. This parameter was previously mainly used to differentiate between causes of anemia. Recent, many reports have illustrated that the level of RDW may be closely associated with high morbidity and mortality, and have shown that it is an independent predictor of high morbidity and mortality in various types of malignancy, diabetes, and cardiovascular, thromboembolic, renal, liver and inflammatory diseases [5–10].

In some clinical settings involving blood loss, prediction of subsequent or concealed hemorrhage is very important. In the past, physicians paid no attention to the association between hemorrhagic loss and the RDW. However, recent studies in cardiovascular disease have shown that RDW has an independent, linear relationship with major bleeding after post-percutaneous coronary intervention [11]. In a trauma setting, a study by Paulus EM *et al*. analyzed the RDW and the requirement for massive transfusion in 3994 trauma patients, and showed a strong relationship between elevated RDW and serious blood loss after traumatic injury [13]. A possible explanation for this is that elevation of the RDW can reflect the dynamic hematologic response to large or subsequent blood loss.

Before the study, we hypothesized that increased RDW may be associated with high-risk UGIB because of the relationship discussed above between dynamic hematologic responses to major or ongoing bleeding and an elevated RDW. In multivariable analysis including previous known risk factors, such as abnormal vital signs, the presence of melena or syncope, underlying comorbidities, and a lower Hg or high BUN, a high RDW showed a strong independent association with high-risk UGIB after adjustment for the other risk factors. More interesting is that the incidence of high-risk UGI is proportional to the RDW level.

For UGIB, it is important to discriminate those high-risk patients who may have uncontrolled bleeding resulting in high morbidity and mortality and the low-risk patients who may be safely discharged home with close and early follow-up [4]. For more accurate and objective risk stratification in emergency department, multiple severity-scoring systems, including the PRS and GBS, have been designed [14,15]. Considering the complexity of the scoring systems compared with the easy accessibility, convenience and low cost of obtaining the RDW, measuring RDW level may be an attractive method to predict high-risk UGIB patients. However, despite its independent dose-dependent relationship with high-risk UGIB, the RDW value alone did not show sufficient discriminatory power in ROC analysis compared with the GBS scoring system for predicting high-risk UGIB.

Nevertheless, the current study identified a beneficial role of RDW in risk stratification for UGIB patients. A predictive scoring model combined with RDW may improve the power to discriminate risk populations. First, for the population with lower GBS (6 or less) or PRS (2 or less), who were thought to have a lower chance of high-risk UGIB [4], RDW can be used as an adjuvant tool. Some patients in this population with lower GBS or PRS system may require treatment and suffer serious morbidity or mortality. Our sub-analysis of the patients with lower scores for both GBS and PRS showed that patients with lower RDW had a lower incidence of high-risk UGIB than the patients with higher RDW. After calculation of the predictive score, a secondary assessment using the RDW level may be helpful to improve the accuracy of risk stratification. Second, our study evaluated the efficacy of the addition of RDW scores derived from our multivariable analysis to the GBS and PRS scoring systems. In ROC analysis to discriminate high-risk UGIB patients, the addition of the RDW score resulted in higher AUC, sensitivity and specificity than using the individual scoring systems. The combination of the RDW score with the PRS score increased the AUC from 0.715 to 0.749. Otherwise, its combination with the GBS system did not show statistical increases in the discriminating power of risk stratification. The GBS system had an inherently stronger power of discrimination than the PRS score; thus, adding the effect of RDW scoring to GBS system may be not so effective in improving discriminating power. Otherwise, the RDW combination model seemed to be more useful in the PRS system for improving the discrimination of high risk in UGIB.

It is difficult in accepting a RDW as simply a predictor of high-risk in UGIB patients who is prone to continuous bleeding without definite pathologic link. Elevations of RDW are often observed in older or populations who had extensive comorbidities. Erythropoietin is known to be the main determinant of RDW. It has been clearly demonstrated that an increase in RDW is influenced by abnormal Erythropoietin production and hypo-functionality of the Erythropoietin response [16,17]. In addition, aging, African ethnicity, strenuous exercise and pregnancy can increase the RDW level physiologically [18–20]. In pathologic conditions, including cell damage by oxidative stress, inflammatory reactions, increased erythrocyte fragmentation, and nutritional deficiency can lead bilologic and metabolic imbalances contributing to increase anisocytosis. This phenomenon is commonplace in various human diseases, including malignancy, cardiovascular disorder, inflammation, liver failure, renal failure and other chronic disease. Above facts are plausible explanations of the strong association between an elevated RDW and poor outcomes in various diseases [5]. High-risk UGIB is mainly associated with massive or subsequent bleeding. Considering that UGIB is a pathologic process involving inflammatory and thrombotic actions in vessels, a possible mechanism for its association with elevated RDW may be the suppression of erythrocyte maturation by inflammatory cytokines [21–22].

This study had several limitations. First, the study population was small. The data were collected from a single tertiary hospital, meaning that the results may have limited generalizability. Second, our study population had a different distribution of causes of UGIB from those seen in Western or other countries. In Korea, variceal bleeding is common because of the high incidence of liver disease resulting from viral hepatitis B and chronic alcoholism. Third, our study population had a higher incidence of high-risk UGIB than those in previous studies. Our study comprised patients from the emergency department of a tertiary hospital; therefore, they may have had more severe disease than patients in smaller emergency departments or primary care facilities. In addition, the high proportion of patients with varix bleeding, which demands more therapeutic intervention, may have contributed to the high incidence of high-risk patients in our study.

## Conclusion

For UGIB patients, a high RDW (≥ 14.5%) was strongly associated with high-risk UGIB. In practice, the combination of RDW with the GBS or PRS scoring indexes in patients with UGIB may increase the accuracy of the identification of low-risk patients and improve the discriminatory power of risk stratification.

## Supporting information

S1 Fig Receiver operating characteristics curves comparing the Area under curve between the PRS system and the PRS plus RDW. (TIF)

S2 Fig Receiver operating characteristics curves comparing the Area under curve between the GBS system and the GBS plus RDW. (TIF)

S1 File All anonymous research data. (XLSX)
