# Supplementary figures and images for: Red cell distribution width as a novel marker for predicting high-risk from upper gastro-intestinal bleeding patients
Source: PLoS One. 2017 Nov 2;12(11):e0187158. doi: 10.1371/journal.pone.0187158 (PMC5667835; doi:10.1371/journal.pone.0187158)

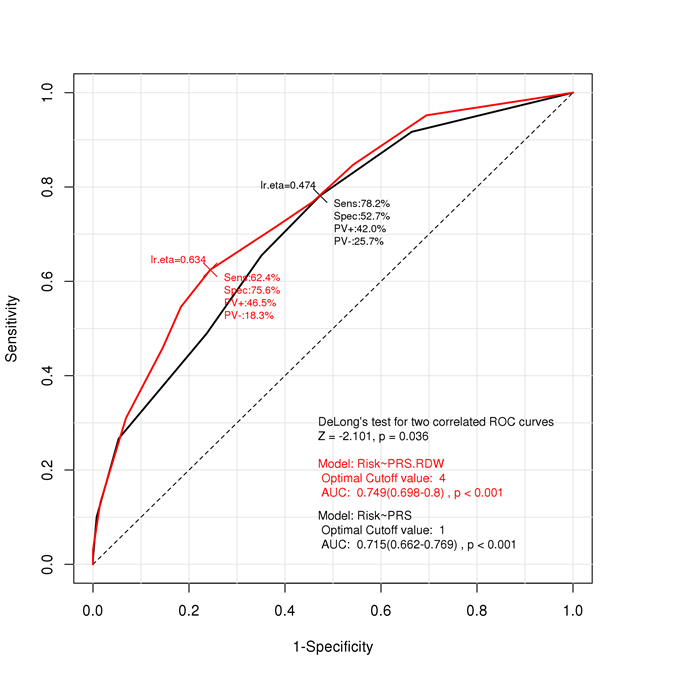

Supplement: S1 Fig — (TIF) [file pone.0187158.s001.tif]

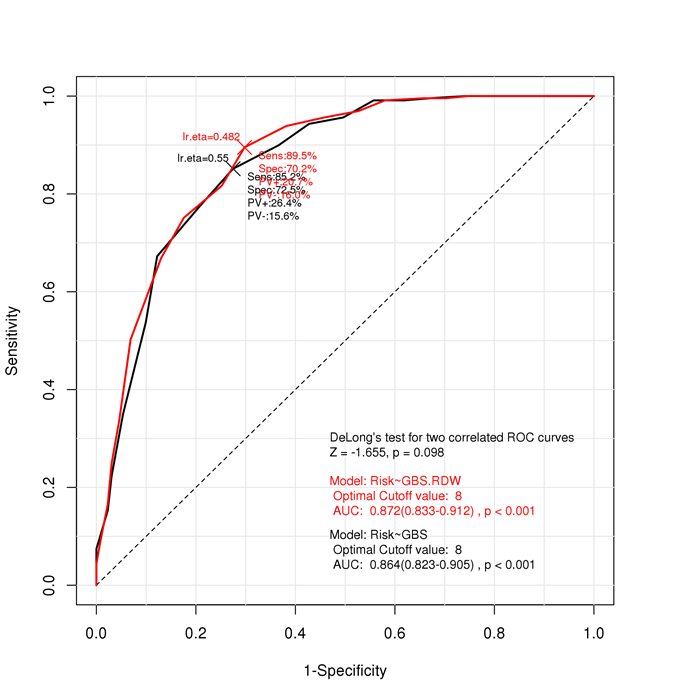

Supplement: S2 Fig — (TIF) [file pone.0187158.s002.tif]
